# Enhanced Anticancer Efficacy of Chemotherapy by Amphiphilic Y-Shaped Polypeptide Micelles

**DOI:** 10.3389/fbioe.2021.817143

**Published:** 2021-12-31

**Authors:** Cong Hua, Yi Zhang, Yuanhao Liu

**Affiliations:** Department of Neurosurgery, The First Hospital of Jilin University, Changchun, China

**Keywords:** polypeptide, polymer topology, micelle, controlled drug delivery, cancer therapy

## Abstract

Although the treatment modalities of cancers are developing rapidly, chemotherapy is still the primary treatment strategy for most solid cancers. The progress in nanotechnology provides an opportunity to upregulate the tumor suppression efficacy and decreases the systemic toxicities. As a promising nanoplatform, the polymer micelles are fascinating nanocarriers for the encapsulation and delivery of chemotherapeutic agents. The chemical and physical properties of amphiphilic co-polymers could significantly regulate the performances of the micellar self-assembly and affect the behaviors of controlled release of drugs. Herein, two amphiphilic Y-shaped polypeptides are prepared by the ring-opening polymerization of cyclic monomer l-leucine *N*-carboxyanhydride (l-Leu NCA) initiated by a dual-amino-ended macroinitiator poly(ethylene glycol) [mPEG-(NH_2_)_2_]. The block co-polypeptides with PLeu_8_ and PLeu_16_ segments could form spontaneously into micelles in an aqueous solution with hydrodynamic radii of 80.0 ± 6.0 and 69.1 ± 4.8 nm, respectively. The developed doxorubicin (DOX)-loaded micelles could release the payload in a sustained pattern and inhibit the growth of xenografted human HepG2 hepatocellular carcinoma with decreased systemic toxicity. The results demonstrated the great potential of polypeptide micellar formulations in cancer therapy clinically.

## Introduction

Chemotherapy is still an essential modality for the treatments of most solid cancers, although a variety of emerging strategies have been developed in the past few decades (Neoptolemos et al., 2018; Bukowski et al., 2020; Lu et al., 2020; Zheng et al., 2020; Zhao et al., 2021). However, the low water solubility, instability, short circulation period, and poor selectivity to tumor tissue of the mainstream small-molecule chemotherapeutic drugs in the clinic restrict the applicable diseases, reduce the anticancer efficacy, and even induce severe side effects (Steinbrueck et al., 2020; Zheng et al., 2021). The development of nanotechnology and the preparation of various organic and inorganic nanoparticles provide a robust tool for the controlled delivery of small-molecule chemotherapeutic drugs (Jiang et al., 2019; Sun et al., 2019; Jiang et al., 2020; Kim et al., 2020; Thomas et al., 2020; Zhang et al., 2020). Among them, the micelles self-assembled from the amphiphilic polymers are attracting increasing attention in the controlled delivery of chemotherapeutic drugs because of their controlled sizes, morphologies, stability, stimulus responsiveness, high drug loading efficiency, and targeted drug delivery behaviors (Zeinali et al., 2020; Bai et al., 2021; Rajes et al., 2021; Wei et al., 2021).

The chemical structures and physical properties of amphiphilic polymers could always significantly regulate the behavior of self-assembly and the performances of obtained micelles (Danafar et al., 2018; Saravanakumar et al., 2018; Brega et al., 2019). The length of hydrophilic and hydrophobic blocks and their ratio are the main factors regulating the sizes, shape, and stability of the polymer micelles and even significantly influence their drug loading properties (Li et al., 2013; Zhang et al., 2013). Typically, longer hydrophobic polymer moieties correspond to smaller sizes, more compact cores, upregulated drug loading capability, and more constant drug release (Hussein and Youssry, 2018).

In addition, the topology of polymers is another critical factor for changing the behavior of self-assembly of polymers and properties of micelles (Li et al., 2013; Yang et al., 2017). The nonlinear amphiphilic polymers, including the graft, Y-shaped, dumbbell-shaped, and even star ones, always induce more stable self-assembled micellar nanoparticles compared with the linear amphiphilic polymers with the same components (Ding et al., 2011; Li et al., 2012; Li et al., 2013; Yang et al., 2021). Therefore, the micelles based on the nonlinear amphiphilic polymers exhibited more promising applications in controlled drug delivery for cancer therapy (Shi et al., 2018; Perin et al., 2021).

In this study, a kind of Y-shaped amphiphilic block co-polymers of methoxy poly(ethylene glycol) (mPEG) and poly(l-leucine) (PLeu) was developed to form spontaneously into micelles and deliver chemotherapeutic agent doxorubicin (DOX) in a controlled manner. PLeu was used as a typical polypeptide segment in this study, and other hydrophobic polypeptides, such as polyglycine, polyvaline, and polyphenylalanine, could also be used as components of amphiphilic block co-polymers for effective drug loading and controlled release. The block co-polypeptides were prepared by the ring-opening polymerization (ROP) of l-leucine *N*-carboxyanhydride (l-Leu NCA) initiated by a dual-amino-ended macroinitiator poly(ethylene glycol) [mPEG-(NH_2_)_2_] (Wang et al., 2019b; Song et al., 2019; Liu et al., 2020). The co-polymer could form into spherical micellar nanoparticles, which exhibited excellent drug encapsulation and release behaviors. The loaded polypeptide micelles showed fascinating tumor growth inhibition efficacy, indicating their great potential for anticancer application clinically (Deng et al., 2020; Zheng et al., 2021).

## Materials and Methods

The materials, synthesis of methoxy poly(ethylene glycol)-(poly (l-leucine))_2_; characterizations, preparation, and characterization of DOX-loaded co-polypeptide micelles; and DOX release *in vitro* are described in the Supplementary Material in detail.

### *In Vitro* Biocompatibility Toward L929 Cells and Cytotoxicity Toward HepG2 Cells

The biocompatibility of mPEG-(PLeu)_2_ against mouse fibroblast L929 cells and the cytotoxicity of mPEG-(PLeu)_2_/DOX toward human hepatocellular carcinoma HepG2 cells were assessed by a typical methyl thiazolyl tetrazolium (MTT) technique *in vitro*. The selected cells were inoculated into tissue culture plates (TCPs) with 96 wells, with 7,000 cells in a well and dispersed in 200.0 μl of Dulbecco’s modified Eagle’s medium (DMEM) with fetal bovine serum (FBS) and antibiotics. At 24 h post-inoculation, the original incubation DMEM was removed, and the solutions of mPEG-(PLeu)_2_ micelles at a concentration from 1.6 to 100.0 μg ml^−1^ or mPEG-(PLeu)_2_/DOX at a concentration from 0.16 to 10.0 μg ml^−1^ in DMEM was supplemented. In 72 h post-co-incubation, the cell viability was determined by an MTT technique. The absorbances of the MTT co-incubated cell solutions in dimethyl sulfoxide (DMSO) were tested at 490 nm on a microplate reader (Bio-Rad 680, Bio-Rad Laboratories, Hercules, CA, USA). The cell viability is assessed in Eq. 1.

$$Cell\;viability\;\left(\%\right)=\frac{A_{Sample}}{A_{Control}}\times 100\%$$ (1)

In Eq. 1, *A*_Sample_ and *A*_Control_ represent the absorbances of the corresponding groups.

### *In Vivo* Tumor Suppression

The BALB/c nude mice (female, 6 weeks old) were bought from Beijing Vital River Laboratory Animal Technology Co., Ltd. (Beijing, China). The suppression efficacy of tumor growth by various DOX formulations was evaluated toward the human HepG2 hepatocellular carcinoma-xenografted BALB/c node mice (Zhao et al., 2013; Wang et al., 2019a). The tumor-bearing mouse model was prepared by injecting subcutaneously 1.0 × 10^6^ HepG2 cells dispersed with 100.0 μl of 0.01 M of phosphate-buffered saline (PBS) to the right anterior limb’s armpit in BALB/c nude mice (5 weeks old, ∼25 g). When the tumor volume reached about 72 mm^3^, the mice were stochastically separated into four groups (*n* = 6). The control of PBS or various formulations of model chemotherapeutic agent DOX, including free DOX, mPEG-(PLeu_8_)_2_/DOX, or mPEG-(PLeu_16_)_2_/DOX, were administrated by the tail-vein injection to treat the tumor-bearing mice. The equivalent DOX dosage was set as 5.0 mg for every kg body weight [mg (kg BW)^−1^]. The chemotherapy was given every 4 days. The tumor volume was assessed every 2 days, and the body weight was detected at the same frequency.

The tumor volume could be assessed according to Eq. 2.

$$Tumor\;volume\;\left(mm^{3}\right)=\frac{L\times W^{2}}{2}$$ (2)

The tumor growth rate was calculated according to Eq. 3.

$$Tumor\;growth\;rate=\frac{Tumor\;volume_{t}}{Tumor\;volume_{0}}$$ (3)

## Results and Discussion

### Synthesis and Characterizations of Methoxy Poly(ethylene glycol)–(Poly(l-leucine))_2_

**SCHEME 1:**
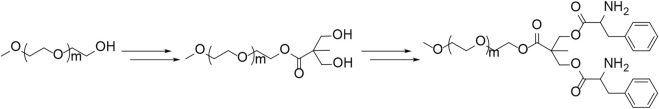
Synthesis route of mPEG-(NH_2_)_2_.

**SCHEME 2:**
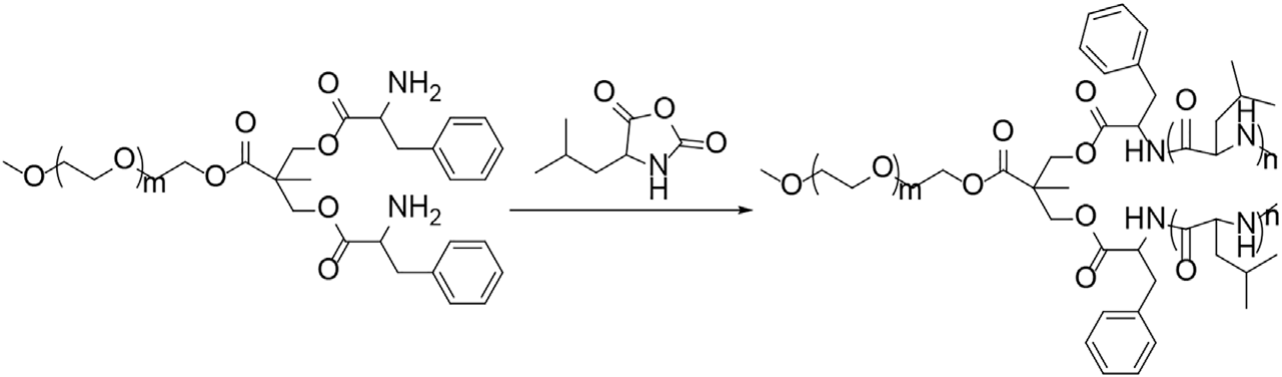
Synthesis pathway of mPEG-(PLeu)_2_.

**FIGURE 1:**
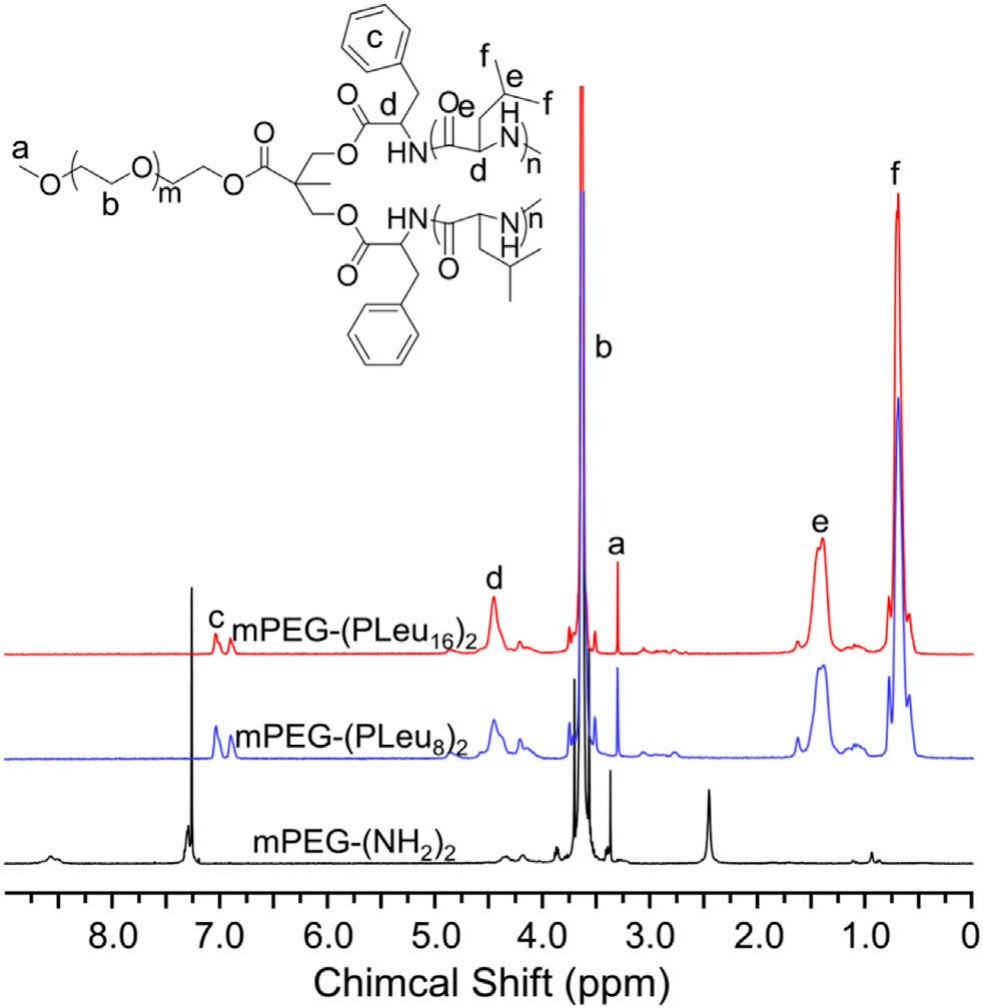
^1^H NMR spectra of mPEG-(NH_2_)_2_, mPEG-(PLeu_8_)_2_, and mPEG-(PLeu_16_)_2_.

**FIGURE 2:**
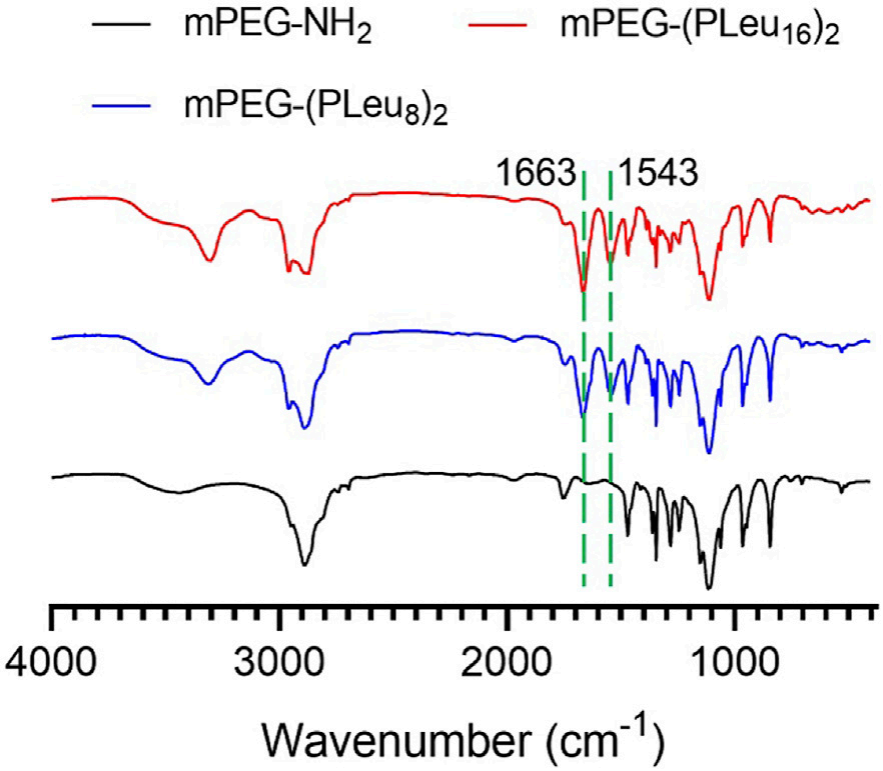
FT-IR spectra of mPEG-(NH_2_)_2_, mPEG-(PLeu_8_)_2_, and mPEG-(PLeu_16_)_2_. FT-IR, Fourier transform IR.

Polypeptides could be prepared by the ROP of amino acid NCA monomers in a controlled manner (Liu et al., 2020; Lv et al., 2020; Vrijsen et al., 2020). In this study, the Y-shaped co-polypeptide mPEG-(PLeu)_2_ was prepared by the ROP of l-Leu NCA with the dual-amino-ended mPEG-(NH_2_)_2_ as a macroinitiator, as shown in Schemes 1 and 2. The successful synthesis of macroinitiator mPEG-(NH_2_)_2_ and the co-polypeptide mPEG-(PLeu)_2_ was demonstrated through the spectrum results of ^1^H NMR and Fourier transform IR (FT-IR). As demonstrated by the results of Figure 1, the appearance of the peaks at 4.45 ppm is attributed to the backbone proton (d, –NHC*H*C(O)–(CH_2_–)), 1.41 ppm is attributed to the methylene protons in the side segment (e, –C*H*_2_C*H*(CH_3_)_2_), and 0.71 ppm belonged to the protons of side methyl groups (f, –CH_2_CH(C*H*_3_)_2_), which proved the successful preparation of mPEG-(PLeu)_2_. The degrees of polymerization (DPs) of PLeu segments were detected to be 8 and 16 based on the area ratios between the resonance peak at 1.41 ppm and that at 3.64 ppm assigned to the protons in methylene segment in the backbone of mPEG (b, –C*H*_2_C*H*_2_O–). Moreover, the successful preparation of mPEG-(PLeu)_2_ was further confirmed by the results of FT-IR, as depicted in Figure 2. The wavenumbers at 1,543 and 1,663 cm^−1^ should be attributed to the stretching vibration of C(O)–NH (ν_C(O)–NH_) and C═O (ν_C═O_), respectively, revealing the formation of the amide bond and the synthesis of PLeu. Moreover, the stretching vibration signal of C–O–C (ν_C–O–C_) demonstrated the appearance of mPEG in the co-polypeptides. Therefore, the results proved the successful synthesis of mPEG-(PLeu_8_)_2_ and mPEG-(PLeu_16_)_2_.

### Preparation and Detection of Doxorubicin-Loaded Co-Polypeptide Micelles *In Vitro*

**FIGURE 3:**
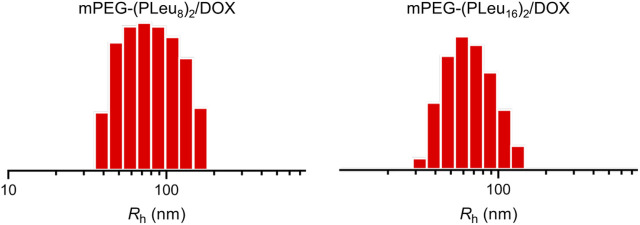
*R*_h_s of mPEG-(PLeu_8_)_2_/DOX and mPEG-(PLeu_16_)_2_/DOX. DOX, doxorubicin.

The micelles from various amphiphilic polymers or co-polymers could be effective nanocarriers for the controlled delivery of anticancer drugs (Ray et al., 2018; Pinyakit et al., 2020). In this work, the chemotherapeutic agent DOX was encapsulated to the cores of micelles of mPEG-(PLeu_8_)_2_ and mPEG-(PLeu_16_)_2_ through nanoprecipitation. The contents of DOX in the micelles were tested by high-performance liquid chromatography (HPLC), and the drug-loading content (DLC) of mPEG-(PLeu_8_)_2_, and mPEG-(PLeu_16_)_2_ micelles were calculated to be 8.75 and 12.94 wt%. The drug-loading efficiency (DLE) of the above micelles were assessed to be 47.95 and 74.32 wt%, respectively. The higher DLC and DLE of mPEG-(PLeu_16_)_2_ micelle resulted from the more hydrophobic property of a longer PLeu block (Chen et al., 2018; Lübtow et al., 2019). As shown in Figure 3, the hydrophobic radii (*R*_h_s) of DOX-loaded co-polypeptide micelles mPEG-(PLeu_8_)_2_/DOX and mPEG-(PLeu_16_)_2_/DOX were detected to be 80.0 ± 6.0 and 69.1 ± 4.8 nm, respectively. The smaller size of mPEG-(PLeu_16_)_2_/DOX also should be assigned to the more hydrophobic performance of PLeu_16_ (Hussein and Youssry, 2018).

**FIGURE 4:**
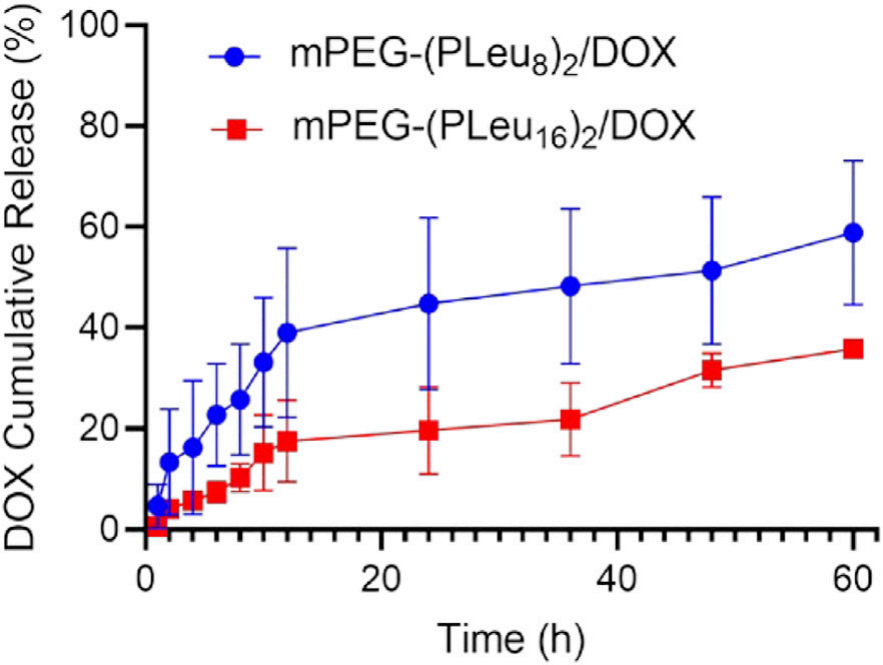
Cumulative DOX release from mPEG-(PLeu_8_)_2_/DOX and mPEG-(PLeu_16_)_2_/DOX in release medium of PBS at pH 7.4. DOX, doxorubicin; PBS, phosphate-buffered saline.

The release behaviors of micelles always affected the therapeutic efficacy of loaded micelles (Wang et al., 2019c). The sustained drug release profiles of mPEG-(PLeu_8_)_2_/DOX and mPEG-(PLeu_16_)_2_/DOX were detected. As depicted in Figure 4, mPEG-(PLeu_8_)_2_/DOX released 58.88% of loaded DOX in the detected 60 h, while mPEG-(PLeu_16_)_2_/DOX released 35.89%. The more compact and hydrophobic core of the micelle always induces a higher drug loading capability and more sustained drug release behavior.

**FIGURE 5:**
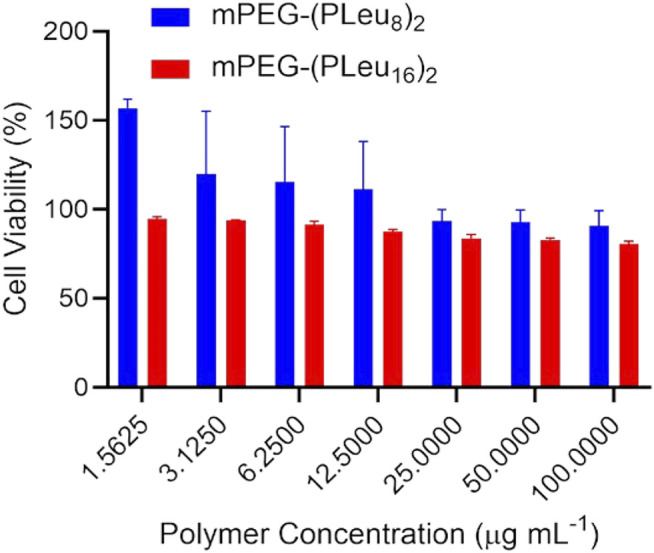
Cytotoxicity of Y-shaped polypeptide micelle toward L929 cells.

The biocompatibility of materials is a critical factor for biomedical applications (Ding et al., 2013; Abbina et al., 2017; Reina et al., 2017). This study detected the biocompatibility of the prepared co-polypeptide micelles of mPEG-(PLeu_8_)_2_ and mPEG-(PLeu_16_)_2_ by an MTT protocol toward L929 cells. As depicted in Figure 5, both the micelles showed excellent biocompatibility at the concentration of 100.0 μg ml^−1^, which was revealed by the high cell viability at above 80%.

**FIGURE 6:**
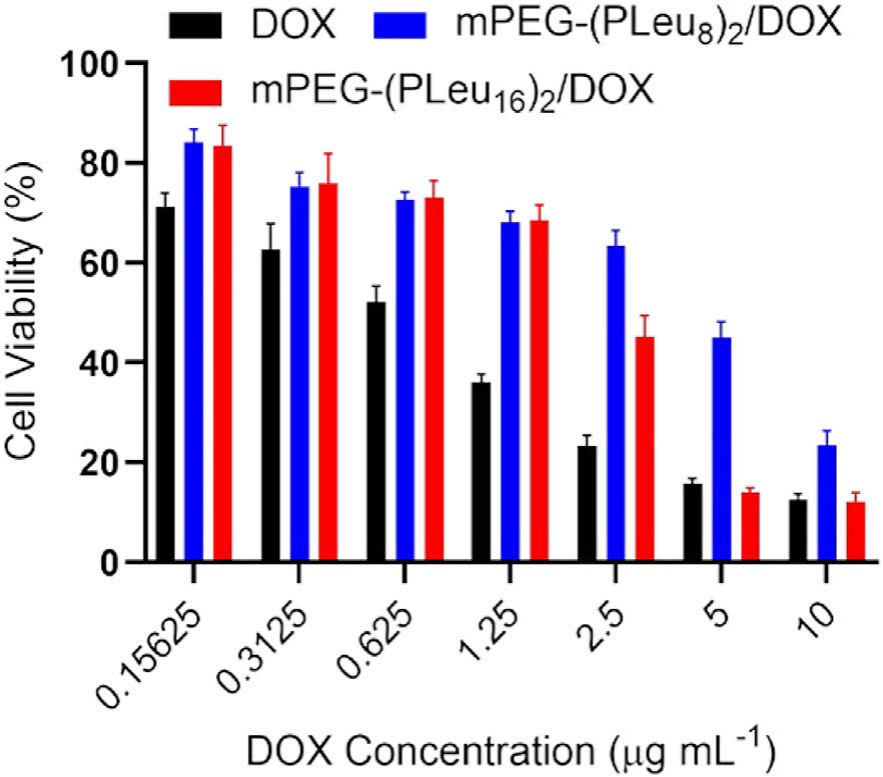
Cell proliferation inhibition efficacy of free DOX, mPEG-(PLeu_8_)_2_/DOX, and mPEG-(PLeu_16_)_2_/DOX toward HepG2 cells. DOX, doxorubicin.

An MTT technique detected the antitumor efficacy of various DOX formulations toward HepG2 cells *in vitro*. As demonstrated by Figure 6, free DOX showed the best inhibition efficiency of cell proliferation as a benefit of its fastest cell entry speed through dispersion. The slightly weaker anticancer effect of DOX-loaded co-polypeptide micelles could be assigned to the slower endocytosis speed by HepG2 cells. In addition, the better anticancer effect of mPEG-(PLeu_16_)_2_ should be attributed to the more sustained release profile of the chemotherapeutic agent in the cells.

### Anticancer Efficacy of Doxorubicin-Loaded Co-Polypeptide Micelles *In Vivo*

The excellent anticancer efficacy of nanoformulations of chemotherapeutic drugs is one of the essential properties for potential clinical application (Ghosh et al., 2021; Zheng et al., 2021). Herein, the anticancer efficacy of PBS as control, free DOX, mPEG-(PLeu_8_)_2_/DOX, and mPEG-(PLeu_16_)_2_/DOX were detected toward human HepG2 hepatocellular carcinoma-xenografted BALB/c node murine model.

**FIGURE 7:**
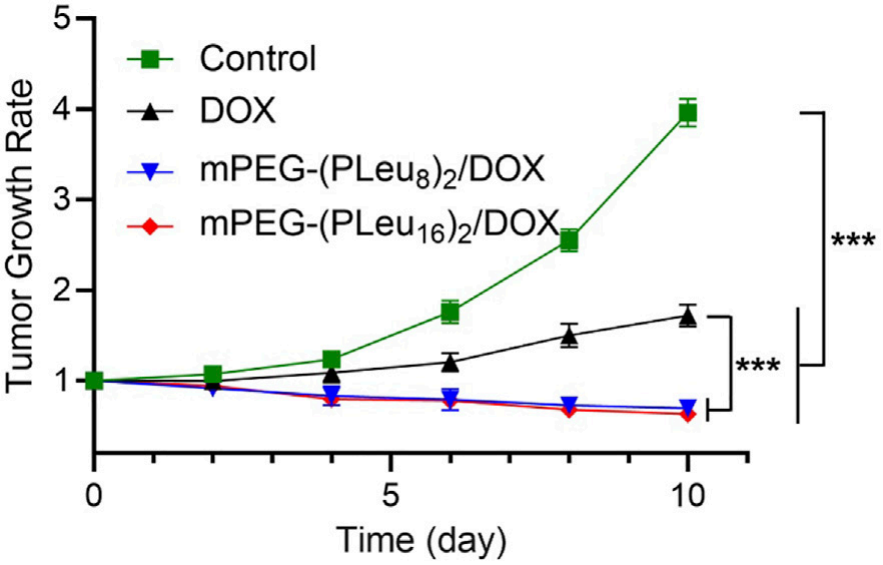
Tumor growth rate of human HepG2 hepatocellular carcinoma-xenografted BALB/c node mice during treatment with PBS as control, free DOX, mPEG-(PLeu_8_)_2_/DOX, or mPEG-(PLeu_16_)_2_/DOX. The data are represented as mean ± SD (*n* = 6; ***p < 0.001). PBS, phosphate-buffered saline; DOX, doxorubicin.

The tumor-bearing mice were constructed by inoculating 1.0 × 10^6^ HepG2 cells dispersed in 100.0 μl of PBS into the right anterior limb’s armpit in a BALB/c nude mouse. As the tumor grew to ∼72 mm^3^ in volume, the model animals were divided into four groups (*n* = 6). On days 1, 4, and 8, the human HepG2 hepatocellular carcinoma-xenografted mice were treated with PBS as control, free DOX, mPEG-(PLeu_8_)_2_/DOX, or mPEG-(PLeu_16_)_2_/DOX three times. The dosage of DOX was set at 5.0 mg (kg BW)^−1^. The tumor volume was monitored every other day. As shown in Figure 7, the tumor growth rates calculated based on the data of tumor volumes decreased to some extent in the groups of mPEG-(PLeu_8_)_2_/DOX and mPEG-(PLeu_16_)_2_/DOX, while it increased to about 3.96 and 1.72 in the PBS as control and free DOX groups, respectively. The best anticancer efficacy should be assigned to the target release of DOX by the micelles of Y-shaped co-polypeptides.

**FIGURE 8:**
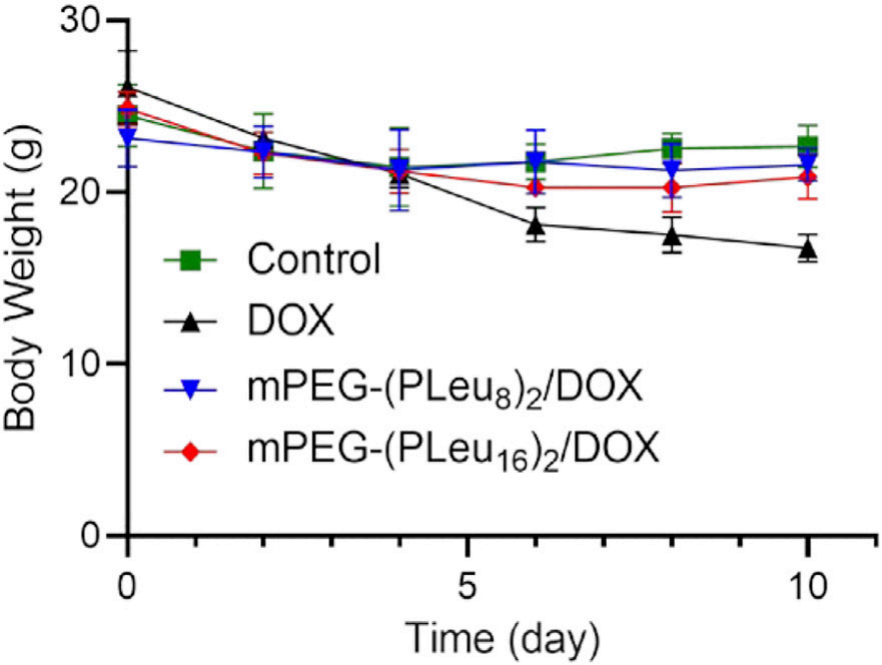
Body weight change of human HepG2 hepatocellular carcinoma-xenografted BALB/c node mouse model during treatment with PBS as control, free DOX, mPEG-(PLeu_8_)_2_/DOX, or mPEG-(PLeu_16_)_2_/DOX. PBS, phosphate-buffered saline; DOX, doxorubicin.

The safety of nanoformulations of chemotherapeutic drugs is another critical factor influencing their potential application in a clinic (Zheng et al., 2021). In this work, the safety of free DOX and DOX-loaded Y-shaped co-polypeptide micelles was revealed by the change of body weight of human HepG2 hepatocellular carcinoma-xenografted mice in the process of treatment. As shown in Figure 8, the body weight decreased significantly after the treatment with free DOX, indicating the toxicity of free DOX *in vivo*. Fortunately, the body weight of mPEG-(PLeu_8_)_2_/DOX and mPEG-(PLeu_16_)_2_/DOX groups was similar to the body weight of the control group, demonstrating their excellent safety *in vivo*. Therefore, the findings confirmed that the Y-shaped polypeptide micelles exhibited great potential for controlled delivery of chemotherapeutic drugs *in vivo*, which benefited the improved anticancer efficacy and reduced systemic toxicity.

## Conclusion

Chemotherapy is still the primary treatment modality for most solid tumors. To overcome the disadvantages of small-molecule drugs, including low water solubility, short circulation time, and low targeting *in vivo*, a variety of nanocarriers were developed. In this study, two Y-shaped co-polypeptides were prepared by the ROP of l-Leu NCA. mPEG-(PLeu)_2_ could self-assemble into micelle and effectively encapsulate and release the model chemotherapeutic drug DOX in a sustained manner. The DOX-loaded Y-shaped co-polypeptide micelles could significantly suppress the growth of HepG2 hepatocellular carcinoma *in vitro* and *in vivo* with reduced systemic toxicity. The results indicated the great potential of the micelles from nonlinear amphiphilic polymers or co-polymers in cancer therapy clinically.

In this study, the human HepG2 hepatocellular carcinoma and DOX are just models to characterize and confirm the advantages of micelles from nonlinear amphiphilic polymers or co-polymers. The developed advanced micelles could be used for the controlled delivery of other therapeutic agents to treat different kinds of cancers and even other diseases.

## Data Availability

The original contributions presented in the study are included in the article/Supplementary Material. Further inquiries can be directed to the corresponding author.
