# Supplementary material for: Enhanced Anticancer Efficacy of Chemotherapy by Amphiphilic Y-Shaped Polypeptide Micelles
Source: Front Bioeng Biotechnol. 2021 Dec 31;9:817143. doi: 10.3389/fbioe.2021.817143 (PMC8758568; doi:10.3389/fbioe.2021.817143)
Supplement: Supplementary file 1 [file DataSheet1.docx]

Supplementary Material

Enhanced Anti-Cancer Efficacy of Chemotherapy by Amphiphilic Y-Shaped Polypeptide Micelles

Cong Hua^*^, Yi Zhang and Yuanhao Liu

Department of Neurosurgery, The First Hospital of Jilin University, 1 Xinmin Street, Changchun 130021, P. R. China

*** Correspondence:**Cong Hua
huacong@jlu.edu.cn

# MATERIALS AND METHODS

## 1.1. Materials

mPEG (number-average molecular weight (*M*_n_) = 5000 g mol^−1^) and methyl thiazolyl tetrazolium (MTT) were purchased from Sigma-Aldrich (Shanghai, P. R. China). 1-Ethyl-3-(3-dimethyl aminopropyl carbodiimide hydrochloride (EDC·HCl), 4-dimethylaminopyridine (DMAP), and *N*,*N*-dimethylformamide (DMF) were purchased from Aladdin Agent Co. (Shanghai, P. R. China). As shown in Scheme 1, the dual-amino-terminated macroinitiator mPEG-(NH_2_)_2_ was synthesized through the condensation reaction between the dual-hydroxyl-terminated mPEG and *t*-Boc-protected L-phenylalanine and the subsequent deprotection (Ding et al., 2011). L-Leu was obtained from GL Biochem. Ltd. (Shanghai, P. R. China), and L-Leu NCA was prepared according to the previously reported protocol (Ulkoski and Scholz, 2018;Grazon et al., 2021a). DMF was dehydrated with calcium hydride (CaH_2_) for 72 h and subsequently distilled in a vacuum. Doxorubicin hydrochloride (DOX·HCl) was purchased from Huafeng United Technology Co. Ltd. (P. R. China). All the other chemicals were obtained from Sigma-Aldrich (Shanghai, P. R. China) and used directly. The deionized water was prepared through Milli-Q water purification equipment (Millipore Co., MA, USA).

Clear 96-well tissue culture polystyrene (TCP) plate was obtained from Corning Costar Co. (Cambridge, MA, USA). Mouse fibroblast cell line L929 and human hepatocellular carcinoma cell line HepG2 were purchased from Shanghai Institute of Biochemistry and Cell Biology, Chinese Academy of Sciences (Shanghai, P. R. China).

Dulbecco's modified Eagle's medium (DMEM) was purchased from Dalian Meilun Biotechnology Co., Ltd. (Dalian, P. R. China). Fetal bovine serum (FBS) was obtained from Tianjin Kangyuan Biotechnology Co., Ltd. (Tianjin, P. R. China). Trypsin-ethylenediaminetetraacetic acid (EDTA) solution and penicillin-streptomycin liquid (penicillin: 10,000.0 U mL^−1^, streptomycin: 10.0 mg mL^−1^) were purchased from Solarbio (Beijing, P. R. China).

The BALB/c nude mice were bought from Beijing Vital River Laboratory Animal Technology Co., Ltd. (Beijing, P. R. China). The animal experiment in this study was approved by the Institutional Animal Care and Use Committee of Jilin University (Changchun, P. R. China).

**1.2. Synthesis of Methoxy Poly(ethylene glycol)-(Poly(L-leucine))_2_**

As shown in Scheme 2, the designed Y-shaped co-polypeptides methoxy poly(ethylene glycol)-(poly(L-leucine))_2_ (mPEG-(PLeu)_2_) was synthesized by the ring-opening polymerization (ROP) of L-Leu NCA with the dual-amino-terminated mPEG-(NH_2_)_2_ as a macroinitiator according to the previously reported protocol (Li et al., 2017;Mostoufi et al., 2019;Grazon et al., 2021b). Typically, the water in mPEG-(NH_2_)_2_ was removed by the azeotropic distillation with toluene at 120 °C. After the complete removal of toluene, the solution of L-Leu NCA in DMF was added. The reaction was performed at room temperature for three days. Then, the mixture was precipitated in diethyl ether. After filtration, the obtained solid product was re-dissolved in DMF and re-precipitated in diethyl ether. The dissolve-precipitation cycle was repeated three times, and the product was dried to constant weight before using.

**1.3. Characterizations**

Proton nuclear magnetic resonance (^1^H NMR) spectra of the macroinitiator mPEG-(NH_2_)_2_ and mPEG-(PLeu)_2_ were detected on a Bruker AV 500 NMR spectrometer (Bruker Biospin Inc., Ettlingen, Germany) in deuterated trifluoroacetic acid (TFA-*d*). Fourier transform infrared (FT-IR) spectra were determined on a Bio-Rad Win-IR instrument (Cambridge, MA, USA) through the potassium bromide approach.

**1.4. Preparation and Characterization of Doxorubicin-Loaded Co-Polypeptide Micelles**

Similar to the standard protocol for drug encapsulation by polymer micelles, the DOX-loaded mPEG-(PLeu)_2_ micelles (mPEG-(PLeu)_2_/DOX) were prepared by nanoprecipitation (Sabra et al., 2018;Sahoo et al., 2019;Zheng et al., 2020). Briefly, the co-polypeptide (20.0 mg) and DOX·HCl (4.3 mg) were dissolved in 2.0 and 0.5 mL of DMF, respectively, and mixed thoroughly. After being stirred at room temperature for 2 h, the mixture was added into 10.0 mL of 0.001 M phosphate-buffered saline (PBS) dropwise. The obtained solution was stirred at room temperature for another 5 h and subsequently dialyzed with deionized water for 24 h to remove the unencapsulated DOX and DMF through a dialysis tube of molecular weight cut-off (MWCO) at 3500 Da. During dialysis, the medium was replaced every 2 h, and mPEG-(PLeu)_2_/DOX were obtained by lyophilization.

To determine the drug-loading content (DLC) and drug-loading efficiency (DLE), mPEG-(PLeu)_2_/DOX was dissolved in DMF, and the content of drug loaded in the micelle was detected by high-performance liquid chromatography (HPLC) (Waters e2695 HPLC system; Waters, Milford, MA, USA). The DLC and DLE of mPEG-(PLeu)_2_/DOX were calculated by Equations (1) and (2).

$\mathrm{DLC}\left( wt\% \right)=\frac{Weight of drug in micelle}{Weight of drug-loaded micelle}\times100\%$ (1)

$\mathrm{DLE}\left( wt\% \right)=\frac{Weight of drug in micelle}{Weight of feeding drug}\times100\%$ (2)

**1.5. Doxorubicin release *in vitro***

The DOX release behaviors of mPEG-(PLeu)_2_/DOX were assessed against 0.01 M PBS at pH 7.4. Briefly, 2.0 mg of mPEG-(PLeu)_2_/DOX was dissolved in 10.0 mL of PBS and transferred into a dialysis tube with MWCO of 3500 Da. The laden dialysis tube was put into 100.0 mL of PBS to start the release assay in the conditions of 75 rpm shock and 37 °C, mimicking the circulation environments *in vivo*. At a predetermined point in time, 2.0 mL of release medium was taken out, and an equal volume of PBS was added. HPLC detected the cumulative DOX release.

**1.6. Statistical Analysis**

All the statistical data were repeated at least three times and represented as mean ± standard deviation (SD). Statistical analysis was performed by GraphPad Prism 8.0.1 for Windows. **P* < 0.05 was recognized as a significant difference, and ***P* < 0.01 and ***P < 0.005 were considered as a highly significant difference.

# REFERENCES

Ding, J., Zhuang, X., Xiao, C., Cheng, Y., Zhao, L., He, C., Tang, Z., and Chen, X. (2011). Preparation of photo-cross-linked pH-responsive polypeptide nanogels as potential carriers for controlled drug delivery. *Journal of Materials Chemistry* 21**,** 11383-11391.

Grazon, C., Salas-Ambrosio, P., Antoine, S., Ibarboure, E., Sandre, O., Clulow, A.J., Boyd, B.J., Grinstaff, M.W., Lecommandoux, S., and Bonduelle, C. (2021a). Aqueous ROPISA of α-amino acid N-carboxyanhydrides: polypeptide block secondary structure controls nanoparticle shape anisotropy. *Polymer Chemistry*.

Grazon, C., Salas-Ambrosio, P., Antoine, S., Ibarboure, E., Sandre, O., Clulow, A.J., Boyd, B.J., Grinstaff, M.W., Lecommandoux, S., and Bonduelle, C. (2021b). Aqueous ROPISA of α-amino acid N-carboxyanhydrides: polypeptide block secondary structure controls nanoparticle shape anisotropy. *Polymer Chemistry* 12**,** 6242-6251.

Li, S.-L., Wang, Y., Zhang, J., Wei, W., and Lu, H. (2017). Targeted delivery of a guanidine-pendant Pt(iv)-backboned poly-prodrug by an anisamide-functionalized polypeptide. *Journal of Materials Chemistry B* 5**,** 9546-9557.

Mostoufi, H., Yousefi, G., Tamaddon, A.-M., and Firuzi, O. (2019). Reversing multi-drug tumor resistance to Paclitaxel by well-defined pH-sensitive amphiphilic polypeptide block copolymers via induction of lysosomal membrane permeabilization. *Colloids and Surfaces B: Biointerfaces* 174**,** 17-27.

Sabra, S.A., Elzoghby, A.O., Sheweita, S.A., Haroun, M., Helmy, M.W., Eldemellawy, M.A., Xia, Y., Goodale, D., Allan, A.L., and Rohani, S. (2018). Self-assembled amphiphilic zein-lactoferrin micelles for tumor targeted co-delivery of rapamycin and wogonin to breast cancer. *European Journal of Pharmaceutics and Biopharmaceutics* 128**,** 156-169.

Sahoo, S., Kayal, S., Poddar, P., and Dhara, D. (2019). Redox-Responsive Efficient DNA and Drug Co-Release from Micelleplexes Formed from a Fluorescent Cationic Amphiphilic Polymer. *Langmuir* 35**,** 14616-14627.

Ulkoski, D., and Scholz, C. (2018). Impact of Cationic Charge Density and PEGylated Poly(amino acid) Tercopolymer Architecture on Their Use as Gene Delivery Vehicles. Part 1: Synthesis, Self-Assembly, and DNA Complexation. *Macromolecular Bioscience* 18**,** 1800108.

Zheng, P., Liu, Y., Chen, J., Xu, W., Li, G., and Ding, J. (2020). Targeted pH-responsive polyion complex micelle for controlled intracellular drug delivery. *Chinese Chemical Letters* 31**,** 1178-1182.
